# *circPVT1* promotes silica-induced epithelial-mesenchymal transition by modulating the miR-497-5p/TCF3 axis

**DOI:** 10.7555/JBR.37.20220249

**Published:** 2024-03-26

**Authors:** Siyun Zhou, Yan Li, Wenqing Sun, Dongyu Ma, Yi Liu, Demin Cheng, Guanru Li, Chunhui Ni

**Affiliations:** 1 Department of Occupational Medical and Environmental Health, Key Laboratory of Modern Toxicology of Ministry of Education, Center for Global Health, School of Public Health, Nanjing Medical University, Nanjing, Jiangsu 211166, China; 2 Biomedical Publications Center, Nanjing Medical University, Nanjing, Jiangsu 211166, China; 3 Gusu School, Nanjing Medical University, Nanjing, Jiangsu 211166, China

**Keywords:** silicosis, epithelial-mesenchymal transition, *circPVT1*, miR-497-5p, TCF3

## Abstract

Epithelial-mesenchymal transition (EMT) is a vital pathological feature of silica-induced pulmonary fibrosis. However, whether circRNA is involved in the process remains unclear. The present study aimed to investigate the role of *circPVT1* in the silica-induced EMT and the underlying mechanisms. We found that an elevated expression of *circPVT1* promoted EMT and enhanced the migratory capacity of silica-treated epithelial cells. The isolation of cytoplasmic and nuclear separation assay showed that *circPVT1* was predominantly expressed in the cytoplasm. RNA immunoprecipitation assay and RNA pull-down experiment indicated that cytoplasmic-localized *circPVT1* was capable of binding to miR-497-5p. Furthermore, we found that miR-497-5p attenuated the silica-induced EMT process by targeting transcription factor 3 (TCF3), an E-cadherin transcriptional repressor, in the silica-treated epithelial cells. Collectively, these results reveal a novel role of the *circPVT1*/miR-497-5p/TCF3 axis in the silica-induced EMT process in lung epithelial cells. Once validated, this finding may provide a potential theoretical basis for the development of interventions and treatments for pulmonary fibrosis.

## Introduction

Silicosis is one type of pneumoconiosis, which is caused by a history of long-term inhalation of crystalline silicon dioxide or silica^[1]^. Despite a wide range of measures to control silica exposure in workers, silicosis remains a major occupational disease worldwide^[2]^. The pathogenesis of silicosis is complex and involves persistent epithelial cell injury and consequent epithelial-mesenchymal transition (EMT) in the early stage^[3]^. EMT is a dynamic and reversible process that has been proved necessary for pulmonary fibrosis, during which epithelial cells lose cell-cell adhesion and apical-basal polarity while obtaining mesenchymal characteristics^[4]^. Multiple factors participate in the EMT process, such as transcription factors, alternative splicing, signaling pathways, and post-transcriptional regulation^[5–6]^. However, the molecular mechanisms underlying EMT in silicosis remain to be further investigated.

Circular RNAs (circRNAs), a relatively novel class of non-coding RNAs (ncRNAs), exhibit biological stability, species conservation, and tissue specificity^[7]^. With the growing development of high-throughput sequencing technology and bioinformatics^[8]^, circRNAs have attracted considerable interest because of their unique covalently closed structure and diverse biological functions, especially in pulmonary fibrosis^[9]^. We previously found that circRNA *CDR1as* promoted the EMT process during silicosis through its inhibitory effects on the miR-7/TGFBR2 axis^[10]^. *circPVT1* (hsa_circ_0001821) originates from a genomic locus on chromosome 8q24, derives from a back-splicing event, contains 410 nucleotides, and has been verified in various diseases^[11]^. RNA-sequencing results indicated that *circPVT1* was a downregulated transcript in senescent fibroblasts, compared with the proliferating ones, and that silencing *circPVT1* reversed the proliferative phenotype^[12]^. In medullary thyroid cancer and breast cancer, *circPVT1* knockdown suppressed the EMT process and cancer cell proliferation, migration, and invasion by targeting the miR-455-5p/CXCL12/CXCR4 pathway and miR-204-5p, respectively^[13–14]^. As for fibrotic diseases, *circPVT1* knockdown repressed the levels of transforming growth factor-beta 1 (TGF-β1), α-smooth muscle actin (α-SMA), and connective tissue growth factor induced by hypoxia, thus ameliorating the development and progression of bladder fibrosis^[15]^, but the role of *circPVT1* in the silica-induced pulmonary fibrosis remains to be investigated.

MicroRNAs (miRNAs) are involved in the transcriptional and post-transcriptional regulation of gene expression, which plays crucial roles in ncRNA networks. Studies have shown that *circPVT1* acts as a competing endogenous RNA (ceRNA) for the miR-497 family in non-small cell lung cancer^[16]^ and head and neck squamous cell carcinoma^[17]^. miR-497-5p, a well-known tumor suppressor, was downregulated in various malignant diseases and was correlated with the EMT process^[18–19]^. In the bronchoalveolar fluids from silicosis patients, a significant reduction of miR-497-5p was found by the miRNA profiling^[20]^. Consistently, our previous work confirmed the decreased expression of miR-497-5p in both TGF-β1-activated lung fibroblasts and silica-induced fibrotic lung tissues^[21]^. Nevertheless, little evidence has been shown whether miR-497-5p plays a regulatory role in the silica-induced EMT process in lung epithelial cells.

In the present study, we aimed to explore the role of *circPVT1* in the silica-induced EMT process of lung epithelial cells, thereby providing a potential theoretical basis for developing interventions and treatments for pulmonary fibrosis.

## Materials and methods

### Cell culture and treatment

The human bronchial epithelial (HBE) cells were purchased from the Stem Cell Bank, Chinese Academy of Sciences (Shanghai, China), and grown in Dulbecco's modified Eagle's medium (Gibco, Waltham, MA, USA). The A549 cells were purchased from the American Type Culture Collection (ATCC, Manassas, VA, USA) and grown in BASIC RPMI Medium 1640 basic (Gibco). Both culture media contained 10% (v/v) fetal bovine serum (Gibco), 100 U/mL penicillin, and 100 μg/mL streptomycin (Gibco). Cells were maintained at 37 ℃ with 5% CO_2_.

Silica particles (Cat. #S5631, Sigma-Aldrich, St. Louis, MO, USA), which were silicon dioxide with 99% purity and good monodispersity (size distribution: 99% in 0.5–10 μm, 80% in 1–5 μm), were well ground and autoclaved before experiments. HBE and A549 cells were treated with well-mixed silica suspension for 24 h. Cells were transfected with small interfering RNA (siRNA), plasmid, miRNA mimic, or inhibitor with riboFECTCP Reagent (Cat. #C10511-05, Ribobio, Guangzhou, China) according to the manufacturer's protocol and then were treated with silica suspension for another 24 h if necessary. siRNAs of *circPVT1*, *TCF3*, and negative control were synthesized by GenePharma (Shanghai, China). Plasmids of *TCF3* and control were synthesized by GENEray (Shanghai, China). miR-497-5p mimic, inhibitor, and control were synthesized by RiBoBio.

### RNA preparation and quantitative reverse transcription-PCR (qRT-PCR) analysis

Total RNA from cells was extracted with TRNzol Universal Reagent (TIANGEN, Beijing, China). The PARIS Kit Protein and RNA Isolation System (Invitrogen, Carlsbad, CA, USA) was used to isolate cytoplasmic and nuclear components of cells. A total of 500 ng RNA was reverse-transcribed with HiScript® Ⅱ Q Select RT Supermix (Vazyme, Nanjing, China) or HiScript® Ⅱ Q RT SuperMix for qPCR Kit (Vazyme) into complementary DNA (cDNA). SYBR Green methods (Vazyme) and a real-time PCR system (Roche LightCycler 480 Ⅱ System, Basel, Switzerland) were used to quantify RNA expression. For the RT-PCR analysis, the 2× Taq plus Master Mix (Cat. #P112-01, Vazyme) was used to amplify cDNA. PCR products were separated by 2% agarose gel stained with ethidium bromide. The Gel Doc XR+ gel system (Bio-Rad Laboratories, Hercules, California, USA) was used for imaging. *U6* and *GAPDH* were used as internal controls for miRNA and mRNA, respectively. Primers used for qRT-PCR are listed as follows: *circPVT1*_For, 5′-ATCTCTGCCAACTTCCTTTG-3′, *circPVT1*_Rev, 5′-TCCATCAGGCTCAGAAAATAC-3′; *TCF3*_For, 5′-CAGGTGGTCTTCTATCTTACTCT-3′, *TCF3*_Rev, 5′-CTCAAGCAATAACTTCTCGTC-3′; long non-coding RNA (lncRNA)-*PVT1*_For, 5′-CCTGTGACCTGTGGAGACAC-3′, lncRNA-*PVT1*_Rev, 5′-GCCATCTTGAGGGGCATCTT-3′; *GAPDH*_For, 5′-TCGGAGTCAACGGATTTGGT-3′, *GAPDH*_Rev, 5′-TTCCCGTTCTCAGCCTTGAC-3′; miR-497-5p_RT, 5′-CCTGTTGTCTCCAGCCACAAAAGAGCACAATATTTCAGGAGACAACAGGACAAACC-3′, miR-497-5p_ For, 5′-CGGGCCAGCAGCACACTGT-3′, miR-497-5p_ Rev, 5′-CAGCCACAAAAGAGCACAAT-3′; *U6*-RT, 5′-AACGCTTCACGAATTTGCG-3′, *U6*- For, 5′-GCTTCGGCAGCACATATACTAA-3′, *U6*- Rev, 5′-AACGCTTCACGAATTTGCGT-3′.

### Wound healing assay

After being transfected with siRNA or mimic for 24 h, HBE cells or A549 cells were wounded with 20 µL pipette tips and photographed immediately at 0 h. The wounded cells were then treated with silica for another 24 h and the cell migration images were captured.

### RNase R treatment

Total RNAs (5 μg/group) of HBE or A549 cells were incubated for 15 min with 3 U/μg RNase R and control buffer (Geneseed, Guangzhou, China). Subsequently, the abundance of *circPVT1* and lncRNA-*PVT1* was analyzed by RT-PCR.

### Immunofluorescence (IF) staining

After being fixed with methanol and blocked with 10% BSA for 1 h at room temperature, cells were incubated with E-cadherin primary antibody (A11492, Abclonal, Wuhan, China) at 4 ℃ overnight and then incubated with Cy3-conjugated goat anti-rabbit secondary antibody (Cat. #A0516, Beyotime, Shanghai, China) at room temperature for 1 h. The nuclei were stained with DAPI (Cat. #C1005, Beyotime) for 15 min, and the cells were observed with a fluorescence microscope (Zeiss, LSM700B, Oberkochen, Badenwueberg, Germany).

### RNA pull-down

Pull-down assay was conducted using the Pierce™ Magnetic RNA-Protein PullDown Kit (Cat. #20164, Millipore, Billerica, MA, USA). The biotin-labeled miR-NC probe and miR-497-5p probe were obtained from Ribobio. Briefly, the lysate samples of HBE cells were incubated with miR-NC probe and miR-497-5p probe and streptavidin-coated magnetic beads (Cat. #88816, Thermo Fisher Scientific, Waltham, MA, USA) at 4 ℃ with rotation overnight. After washing with the wash buffer, the RNA complexes were purified and the levels of *circPVT1* or *TCF3* mRNA were measured by qRT-PCR.

### RNA immunoprecipitation (RIP) assay

According to the manufacturer's instructions for the Magna RIP kit (Millipore), about 2 × 10^7^ cells were pelleted and resuspended in lysis buffer. Then, cell lysates were incubated with 5 μg of IgG (Abcam, Cambridge, UK) or AGO2 antibody (Proteintech, Wuhan, China) antibody-magnetic coated beads and rotated at 4 ℃ overnight. The purified co-precipitated RNAs were reverse-transcribed and detected by qRT-PCR.

### Western blotting and antibodies

Protein extracts (80 μg) were separated using 10% SDS-PAGE, transferred onto PVDF membrane (Millipore), and incubated with primary antibodies overnight at 4 ℃. After being washed with TBST for 15 min, membranes were then incubated with a horseradish peroxidase-conjugated secondary antibody (Beyotime) for 1 h at room temperature. After being washed with TBST for 30 min, membranes were imaged using the ChemiDoc XRS+ imaging system (Bio-Rad Laboratories). Image J software was used for densitometry analysis. Primary antibodies: antibody against Fibronectin (1∶1000, Cat. #ab45688, Abcam), E-cadherin (1∶500, Cat. #A11492, Abclonal), vimentin (1∶2000, Cat. #10366-1-AP, Proteintech), α-SMA (1∶1000, Cat. #ab32575, Abcam), TCF3 (1∶1000, Cat. #21242-1-AP, Proteintech), and GAPDH (1∶10000, Cat. #AC033, Abclonal).

### Statistical analysis

GraphPad Prism software (version 6.0) was used for statistical analysis. Student's *t*-test or one-way ANOVA followed by Dunnett's or Tukey's post hoc tests were performed to compare the difference between two or among more groups. Data were presented as mean ± standard deviation. *P* < 0.05 was considered statistically significant.

## Results

### *circPVT1* inhibited the silica-induced EMT process

**Figure 1:**
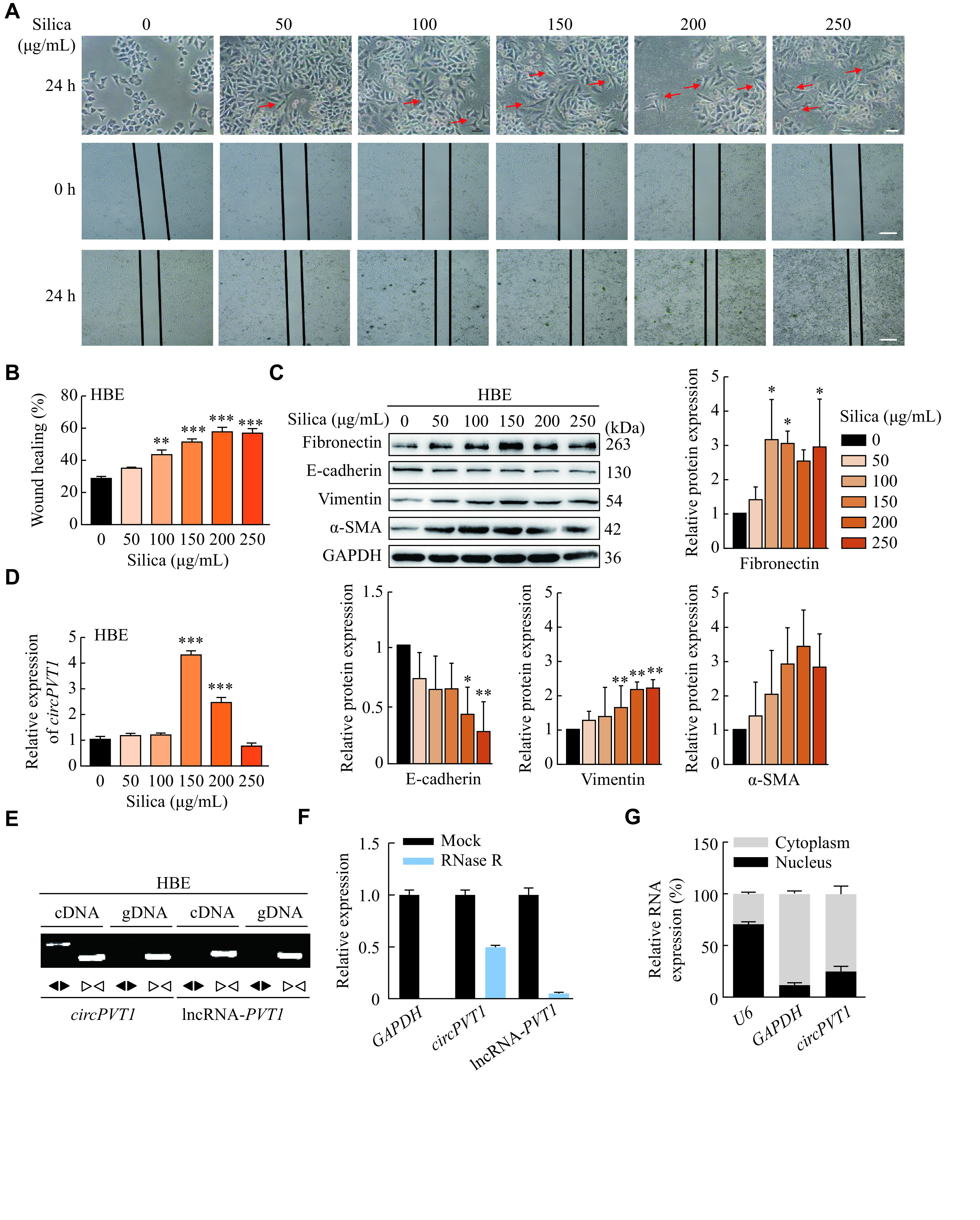
*circPVT1* was involved in the silica-induced EMT process in HBE cells.

To investigate the effect of silica on the EMT process, HBE and A549 cells were treated with different doses of silica particles (0, 50, 100, 150, 200, and 250 μg/mL) for 24 h. We observed a remarkable change in cell morphology from flat and irregularly polygonal epithelial cells with characteristics of easily connected into slices to spindle and irregular triangular mesenchymal cells losing their intercellular connectivity in HBE cells (***Fig. 1A***). The wound healing assay showed that silica treatment significantly enhanced the migratory capacity of HBE cells (***Fig. 1A*** and ***1B***). Moreover, the protein level of epithelial cell marker (E-cadherin) declined, while the protein levels of mesenchymal cell markers (α-SMA, vimentin, and fibronectin) increased in a dose-dependent manner in HBE cells (***Fig. 1C***). Then, we detected *circPVT1* expression in silica-treated epithelial cells and found a nearly four-fold up-regulation of *circPVT1* expression in HBE cells treated with 150 μg/mL silica (***Fig. 1D***). Similar results were observed in silica treated A549 cells, and 100 μg/mL silica treatment showed the highest expression of *circPVT1* (***Supplementary Fig. 1A–1D***, available online). Thus, we chose concentrations of 150 μg/mL and 100 μg/mL silica particles for HBE and A549 cells, respectively, for subsequent experiments.

Before functional exploration of *circPVT1*, we first examined the characteristics of *circPVT1* with a loop structure. Divergent and convergent primers were designed to amplify *circPVT1* in cDNA and genomic DNA of HBE cells. After performing PCR amplification and agarose gel electrophoresis, the amplification product of *circPVT1* was only observed in cDNA using divergent primers (***Fig. 1E***). Because of its covalently closed circular structure, *circPVT1 *was more stable and resistant to RNase R digestion than *lncPVT1* in HBE cells (***Fig. 1F***). Furthermore, nuclear-cytoplasmic separation followed by qRT-PCR analysis suggested that *circPVT1* was predominantly expressed in the cytoplasm of HBE cells (***Fig. 1G***). We also confirmed these characteristics of *circPVT1* in A549 cells (***Supplementary Fig. 1E***–***1G***, available online).

**Figure 2:**
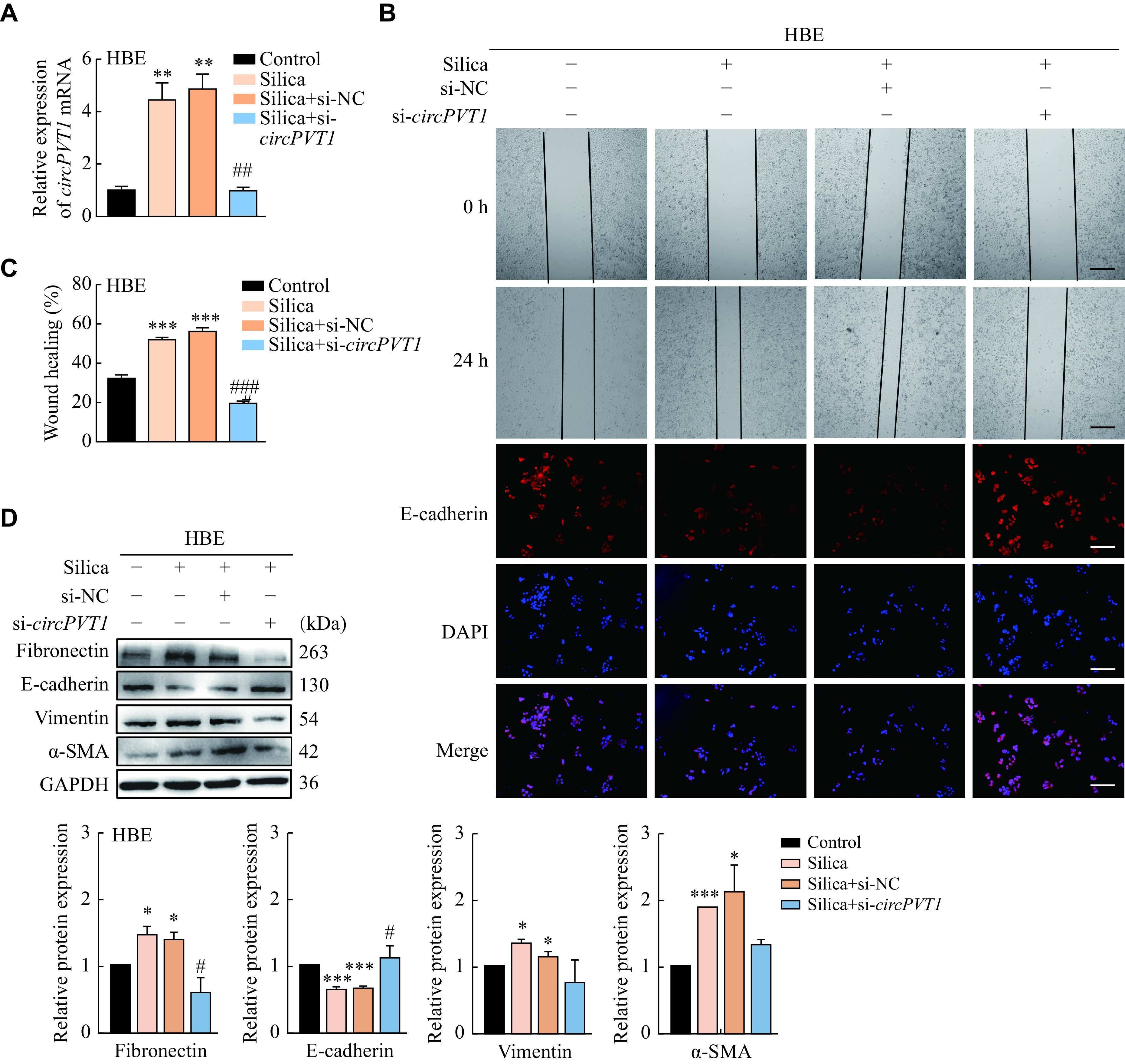
The knockdown of *circPVT1* expression attenuated the silica-induced EMT process in HBE cells.

Considering the significant upregulation of *circPVT1* in silica-treated HBE and A549 cells, we further investigated potential roles of *circPVT1* in epithelial cells. The results of qRT-PCR showed that *circPVT1* knockdown efficiently abrogated the silica-induced upregulation of *circPVT1* in HBE and A549 cells (***Fig. 2A*** and ***Supplementary Fig. 2A*** [available online]). The wound healing assay showed that *circPVT1* knockdown significantly reduced migratory capacity of HBE cells that had been enhanced by silica, and the staining for E-cadherin showed that silencing *circPVT1* along with silica treatment rescued fluorescence intensity of E-cadherin that was inhibited by silica treatment alone in HBE cells (***Fig. 2B*** and ***2C***). Moreover, silica stimulated fibronectin, α-SMA, and vimentin levels but inhibited the expression of E-cadherin, while depletion of *circPVT1* efficiently blocked these processes (***Fig. 2D***). These results were also observed consistently in A549 cells (***Supplementary Fig. 2***), thereby indicating that *circPVT1*, the cytoplasm-enriched circRNA in both HBE and A549 cells, may inhibit the silica-induced EMT process in epithelial cells.

### *circPVT1* acted as a ceRNA for miR-497-5p to regulate the EMT process

**Figure 3:**
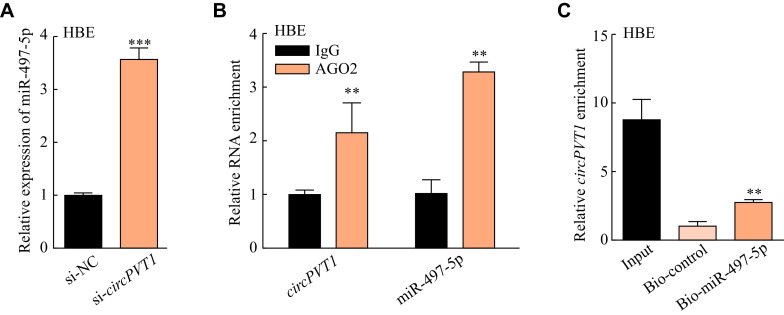
*circPVT1* interacted with miR-497-5p in HBE cells.

Our previous study revealed that *lncPVT1* competitively bound to miR-497-5p to promote fibroblast activation in the silica-induced pulmonary fibrosis^[21]^. Moreover, *circPVT1* was also found to bind to miR-497-5p in head and neck squamous cell carcinoma^[17]^. Thus, we wondered whether *circPVT1* sponges miR-497-5p in the silica-treated epithelial cells. Prediction from RNAhybrid (https://bibiserv.cebitec.uni-bielefeld.de/rnahybrid/) indicated a potential interaction between *circPVT1* and miR-497-5p (***Supplementary Fig. 3A***, available online). The expression of miR-497-5p was increased in the depletion of *circPVT1* (***Fig. 3A*** and ***Supplementary Fig. 3B*** [available online]). Further, the specific AGO2 antibody was able to significantly enrich both endogenous *circPVT1* and miR-497-5p (***Fig. 3B***), and the biotin-labeled miR-497-5p pulled down *circPVT1* in HBE cells (***Fig. 3C***), which both validated the direct interaction of *circPVT1* and miR-497-5p.

**Figure 4:**
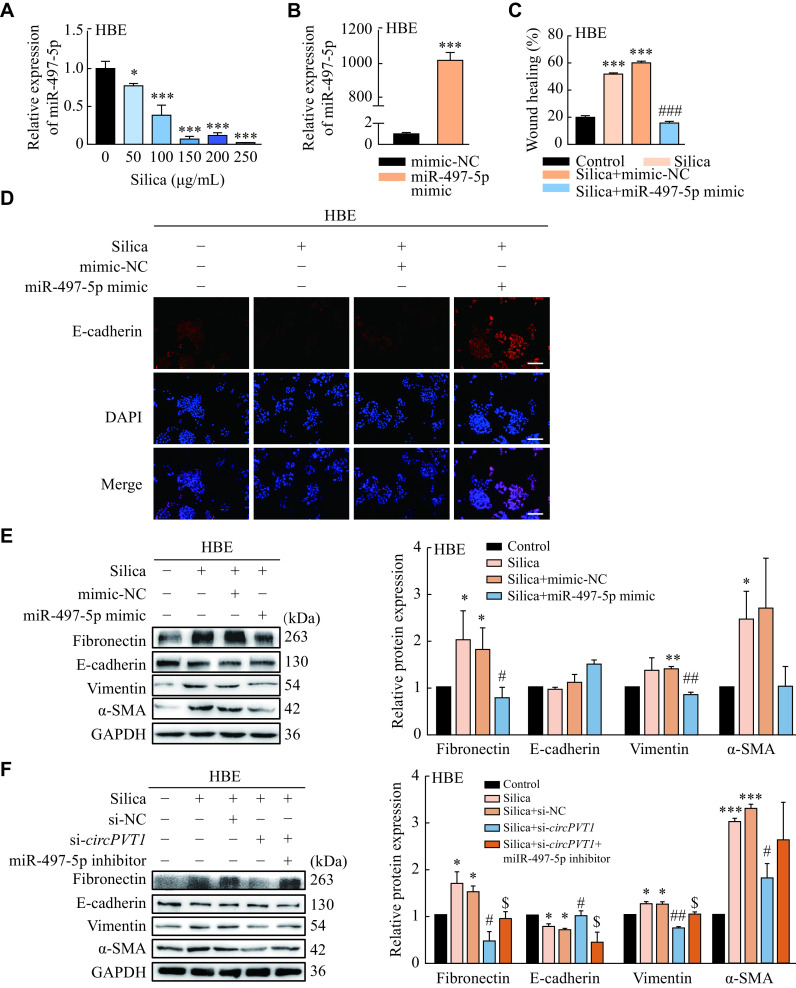
The *circPVT1*/miR-497-5p axis was involved in the silica-induced EMT process in HBE cells.

miR-497-5p decreased significantly in the silica-treated HBE and A549 cells (***Fig. 4A*** and ***Supplementary Fig. 3C*** [available online]). To evaluate whether miR-497-5p participates in the silica-induced EMT process, we used miR-497-5p mimic to treat epithelial cells and observed a decreased migratory capacity of HBE and A549 cells after miR-497-5p overexpression by the wound healing assay (***Fig. 4B*** and ***4C***, ***Supplementary Fig. 3D*** and ***3E*** [available online]). Additional IF assay (staining for E-cadherin) and Western blotting analysis showed that miR-497-5p mimic attenuated the silica-induced EMT process (***Fig. 4D*** and ***4E***, ***Supplementary Fig. 3F*** and ***3G*** [available online]). We then transfected cells with miR-497-5p inhibitor and found that the decreased expression of miR-497-5p abolished the anti-fibrotic and anti-EMT effect of *circPVT1* siRNA (***Fig. 4F*** and ***Supplementary Fig. 4*** [available online]) in the silica-treated HBE and A549 cells. These results indicate that *circPVT1* acted as a ceRNA for miR-497-5p to regulate the silica-induced EMT process.

### miR-497-5p inhibited the EMT process *via* down-regulating TCF3

**Figure 5:**
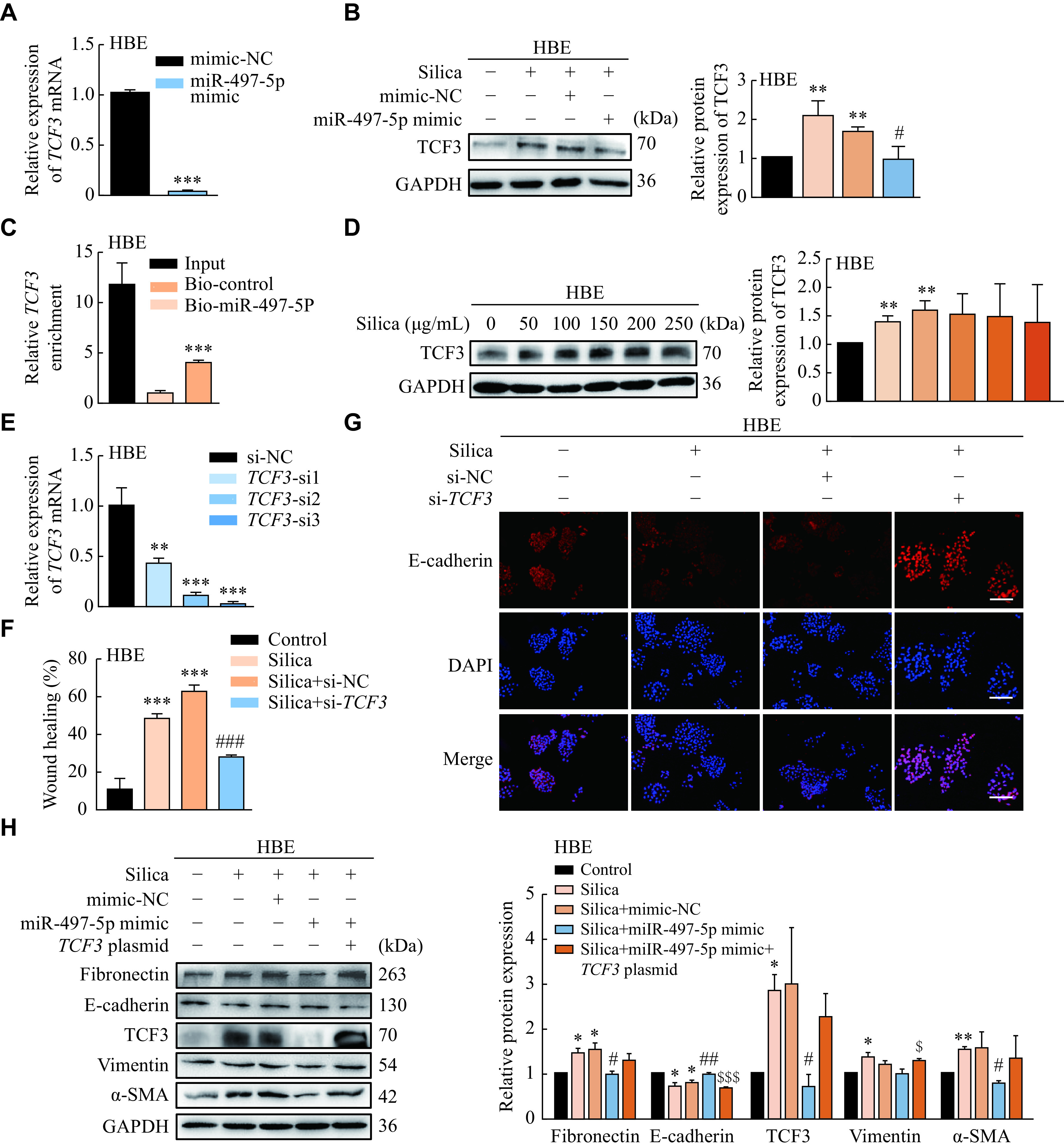
miR-497-5p inhibits the EMT process *via* down-regulating TCF3 expression in HBE cells.

We further predicted potential downstream targets of miR-497-5p in regulating the EMT process using Encyclopedia of RNA Interactomes (ENCORI, https://starbase.sysu.edu.cn/index.php), and identified some complementary binding sites between miR-497-5p and *TCF3* mRNA. TCF3 (also known as E2A), one of the basic helix-loop-helix (bHLH) factors, has been characterized as an E-cadherin transcriptional repressor and triggers the EMT process^[22]^. ***Supplementary Fig. 5A*** (available online) shows the potential binding site of miR-497-5p on the 3′ untranslated region of *TCF3* mRNA. Over-expression of miR-497-5p suppressed both RNA and protein levels of TCF3 in HBE and A549 cells (***Fig. 5A*** and ***5B***, ***Supplementary Fig. 5B*** and ***5C*** [available online]). RNA pull-down assay showed the direct binding relationship between miR-497-5p and *TCF3* mRNA in HBE cells (***Fig. 5C***).

Next, we investigated the function of TCF3 in the silica-induced EMT process. The protein level of TCF3 increased in the silica-treated HBE and A549 cells (***Fig. 5D*** and ***Supplementary Fig. 5D*** [available online]). *TCF3* knockdown enhanced the fluorescence of E-cadherin and weakened the migratory capacity of HBE and A549 cells (***Fig. 5E–5G*** and ***Supplementary Fig. 5E–5G*** [available online]). These results indicate that miR-497-5p may inhibit the silica-induced EMT process *via* down-regulating TCF3.

### TCF3 over-expression abrogated the effects of miR-497-5p mimic in lung epithelial cells

Based on the above presented results, we constructed *TCF3* overexpression plasmid (***Supplementary Fig. 5H*** [available online]). The silica-treated epithelial cells were transfected with miR-497-5p mimic alone or combined with the *TCF3* plasmid. The results of Western blotting assays showed that over-expression of TCF3 partly abolished the anti-fibrotic and anti-EMT effect of miR-497-5p mimic, suggesting that miR-497-5p may prevent the silica-induced EMT process *via* targeting *TCF3* (***Fig. 5H*** and ***Supplementary Fig. 5I*** [available online]).

## Discussion

In 1995, the International Labour Organization and the World Health Organization initiated a global campaign for the elimination of silicosis by 2030. However, silicosis is still a public health concern, partly because of the exposure to modern industrial pollution, the lack of effective therapeutic drugs, and a poor prognosis^[2]^. The mechanism of silicosis has not been fully elucidated, and further in-depth investigation is needed for the disease prevention and treatment.

According to the U.S. Occupational Safety and Health Administration, the permissible exposure limit for respirable SiO_2_ is 0.05 mg/(m^3^·day). Bates *et al*^[23]^ estimated that humans could be exposed to about 1433 mg of respirable silica in 40 years of work (8 h/day for 5 days/week), which would be 8.28 mg as an equivalent lifetime exposure in mice. To build a silicosis model in the laboratory, mice were given a one-off intratracheal perfusion with a silica suspension, and our early exploration found that 2.5 mg was the lowest dose of SiO_2_ to induce pulmonary fibrosis in 6 to 8-week C57BL/6 mice (19–22 g in weight)^[21]^, while some investigators applied higher doses (5.0 mg and 10 mg) of SiO_2_ in their studies^[24–25]^. However, the one-off perfusion process in animal models is quite different from the real-world and long-term exposure of professional populations. Besides, in occupations related to construction, demolition, mining, and fracking, the short-term exposure levels are usually unregulated and frequently surpass the permissible exposure limit^[26]^. In cellular experiments, we treated epithelial cells with different doses of silica particles (0–250 μg/mL) and found that 150 μg/mL (about 14.3 μg/cm^2^) for HBE and 100 μg/mL (about 9.5 μg/cm^2^) for A549 cells were suitable concentrations to induce EMT, as supported by previous studies^[27]^. Referring to data from Schulte *et al*^[28]^, the total alveolar surface area of 3-month C57BL/6 mice is about 550 cm^2^ and the estimated exposure level is 18 μg/cm^2^ after 5.0 mg SiO_2_ treatment. Therefore, the concentrations of SiO_2_ used in the current study may not be excessively high for actual exposure scenarios.

During the EMT process, the expression of *circPVT1* was increased in lung epithelial cells, which has also been reported to be positively associated with tumor cell proliferation, invasion, and radioresistance^[29]^. *circPVT1* derives from the circularization of exon 2 of the plasmacytoma variant translocation 1 gene (*PVT1*), which also encodes lncRNA named *lncPVT1*. Both transcript variants show positive associations with disease progression^[29]^. For instance, over-expressed *lncPVT1* and *circPVT1* promoted the progression of colorectal cancer through the miR-106b-5p/FJX1 and miR-30c-5p/TCF7 axis, respectively^[30–31]^. In pulmonary fibrosis, we found that both *circPVT1* and *lncPVT1* increased in TGF-β1-treated lung fibroblasts (MRC-5 cells), and the basal expression of *lncPVT1* was much higher than that of *circPVT1*. Thus, our previous study focused on the role of *lncPVT1* in fibroblast activation^[21]^. However, *lncPVT1* decreased slightly but *circPVT1* increased significantly in the silica-induced EMT process. Consequently, we investigated the involvement of *circPVT1* in epithelial cells in the current study.

Both *circPVT1* and *lncPVT1* are expressed in the cytoplasm and nucleus. However, given a stabler structure^[32]^, *circPVT1* may regulate cytoplasm-centric miRNA-mRNA interactions more efficiently than *lncPVT1* does^[12]^. Interestingly, both our previous and current investigations have shown that miR-497-5p is a common target of *circPVT1* and *lncPVT1*. Combined with the expression mode of *circPVT1* and *lncPVT1* in lung fibroblasts and epithelial cells as well as the complex process of fibrosis progression, which involves multiple processes, such as EMT and fibroblast activation^[33]^, we speculate that *circPVT1* and *lncPVT1* may play different roles in different cells; that is, *circPVT1* mainly acts on epithelial cells, while *lncPVT1* mainly acts on lung fibroblasts, and both act jointly on miR-497-5p to promote the silica-induced fibrosis. However, whether *circPVT1* and *lncPVT1* synergize through cell-to-cell communication or the mechanisms of interaction with each other in a particular cell type remains unknown and deserves future investigation.

A range of miRNAs were expressed differently in lung tissues of silicosis patients, and results of miRNA profiles (GSE54463) showed a reduced level of miR- 497-5p in a silica-induced mouse model of pulmonary fibrosis^[20,34]^. The participation of miR-497-5p in fibrosis has been investigated, involving myofibroblast differentiation, epithelial EMT, and apoptosis^[35–37]^. Consistently, we observed an anti-EMT effect of miR-497-5p in the silica-treated epithelial cells. In addition, our previous study confirmed that intratracheal instillation of the miR-497-5p agomir reverted the silica-induced pulmonary fibrosis in mice^[21]^, indicating its potential as a therapeutic target. Moreover, EMT intervention has been demonstrated to attenuate pulmonary fibrosis by a growing body of evidence. For example, emodin, an anthraquinone derivative isolated from rhubarb, regulates EMT through the inhibition of both the TGF-β1/Smad3 and NF-κB signaling pathways to prevent alveolar inflammation and apoptotic process^[38]^. Pirfenidone, an oral anti-fibrotic agent approved for idiopathic pulmonary fibrosis treatment, also showed a protective effect by inhibiting EMT in a rat silicosis model^[39]^.

miRNAs bind to complementary sequences of the target mRNA at the 3′ untranslated region to interfere with the structural stability of mRNA and the translation process. *TCF3* mRNA may bind to miR-497-5p as predicted by ENCORI. The *E2A* gene encodes two bHLH transcription factors, E12 and E47 (also called TCF3), which activate transcription by forming homodimers or heterodimers with other bHLH proteins^[22]^. TCF3 protein was identified as a regulator to promote the commitment and differentiation of some lymphocyte lineages^[40]^ and maintain the hematopoietic stem cell pool^[41]^. Its dysregulation has been acknowledged in many disease states, including Burkitt lymphoma, gastric cancer, and breast cancer^[42–44]^. As a potent E-cadherin repressor, TCF-3 activates *SNAIL* transcription by coupling with β-catenin in ovarian endometriosis^[45]^, and the over-expression of TCF3 induces EMT and fibrosis in human renal proximal tubular epithelial cells^[46]^. However, the implication of TCF3 in lung fibrosis is still unknown. Here, we found that TCF3 expression was upregulated in the silica-treated HBE and A549 cells but was inhibited by miR-497-5p mimic. Over-expressed TCF3 partly abolished the anti-EMT effect from miR-497-5p mimic, suggesting TCF3 as a potential anti-fibrotic therapy target.

However, several limitations exist in the present study. The A549 is a pulmonary epithelial cell line derived from human alveolar cell carcinoma, and HBE is a human bronchial epithelial cell line. Here, we used them to replace alveolar type Ⅱ epithelial cells because of their tendency to differentiate. Many studies on pulmonary fibrosis also used these alternatives^[47–48]^. Besides, given the poor conservation of *circPVT1* between humans and mice, there are no animal studies designed to target it. We demonstrated the effect of miR-497-5p, a regulated target of *circPVT1*, *in vivo* previously^[21]^. It would be beneficial to confirm the expression of *circPVT1* in lung tissues or primary lung epithelial cells of silicosis patients, because it would further demonstrate the effect of *circPVT1* in humans.

In summary, the current study has confirmed a novel role of the *circPVT1*/miR-497-5p/TCF3 axis in the silica-induced EMT process in lung epithelial cells. This finding may serve as a potential theoretical basis for the development of intervention and treatment for pulmonary fibrosis.

## SUPPLEMENTARY DATA

Supplementary data to this article can be found online.

## References

[1] (2012). Silicosis. Lancet.

[2] (2020). Silica-related diseases in the modern world. Allergy.

[3] (2015). Fibrosis-A common pathway to organ injury and failure. N Engl J Med.

[4] (2016). Epithelial-mesenchymal transition in tissue repair and fibrosis. Cell Tissue Res.

[5] (2021). Molecular mechanism involved in epithelial to mesenchymal transition. Arch Biochem Biophys.

[6] (2021). Epithelial-mesenchymal transition (EMT): the type-2 EMT in wound healing, tissue regeneration and organ fibrosis. Cells.

[7] (2021). CircRNAs: decrypting the novel targets of fibrosis and aging. Ageing Res Rev.

[8] (2019). Investigation of circular RNAs and related genes in pulmonary fibrosis based on bioinformatics analysis. J Cell Biochem.

[9] (2020). CircRNA TADA2A relieves idiopathic pulmonary fibrosis by inhibiting proliferation and activation of fibroblasts. Cell Death Dis.

[10] (2018). The CDR1as/miR-7/TGFBR2 axis modulates EMT in silica-induced pulmonary fibrosis. Toxicol Sci.

[11] (2017). Circular RNA profile identifies circPVT1 as a proliferative factor and prognostic marker in gastric cancer. Cancer Lett.

[12] (2019). Circular PVT1: an oncogenic non-coding RNA with emerging clinical importance. J Clin Pathol.

[13] (2021). A concise review on the role of CircPVT1 in tumorigenesis, drug sensitivity, and cancer prognosis. Front Oncol.

[14] (2021). circPVT1 regulates medullary thyroid cancer growth and metastasis by targeting miR-455-5p to activate CXCL12/CXCR4 signaling. J Exp Clin Cancer Res.

[15] (2022). Circular RNA Plasmacytoma Variant Translocation 1 (CircPVT1) knockdown ameliorates hypoxia-induced bladder fibrosis by regulating the miR-203/Suppressor of Cytokine Signaling 3 (SOCS3) signaling axis. Bioengineered.

[16] (2019). Circular RNA PVT1 acts as a competing endogenous RNA for miR-497 in promoting non-small cell lung cancer progression. Biomed Pharmacother.

[17] (2017). The oncogenic role of circPVT1 in head and neck squamous cell carcinoma is mediated through the mutant p53/YAP/TEAD transcription-competent complex. Genome Biol.

[18] (2022). CircRTN4 promotes pancreatic cancer progression through a novel CircRNA-miRNA-lncRNA pathway and stabilizing epithelial-mesenchymal transition protein. Mol Cancer.

[19] (2021). miR-497-5p/SALL4 axis promotes stemness phenotype of choriocarcinoma and forms a feedback loop with DNMT-mediated epigenetic regulation. Cell Death Dis.

[20] (2016). Genome-wide analysis of aberrantly expressed microRNAs in bronchoalveolar lavage fluid from patients with silicosis. Ind Health.

[21] (2021). LncRNA-PVT1 activates lung fibroblasts via miR-497-5p and is facilitated by FOXM1. Ecotoxicol Environ Saf.

[22] (2008). E2A proteins: regulators of cell phenotype in normal physiology and disease. Int J Biochem Cell Biol.

[23] (2015). Silica triggers inflammation and ectopic lymphoid neogenesis in the lungs in parallel with accelerated onset of systemic autoimmunity and glomerulonephritis in the lupus-prone NZBWF1 mouse. PLoS One.

[24] (2023). Inhibition of oncogenic src ameliorates silica-induced pulmonary fibrosis *via* PI3K/AKT pathway. Int J Mol Sci.

[25] (2023). A2aR inhibits fibrosis and the EMT process in silicosis by regulating Wnt/β-catenin pathway. Ecotoxicol Environ Saf.

[26] (2021). Rapid induction of pulmonary inflammation, autoimmune gene expression, and ectopic lymphoid neogenesis following acute silica exposure in lupus-prone mice. Front Immunol.

[27] (2021). miR-138 inhibits epithelial-mesenchymal transition in silica-induced pulmonary fibrosis by regulating ZEB2. Toxicology.

[28] (2019). Age-related structural and functional changes in the mouse lung. Front Physiol.

[29] (2021). CircPVT1 promotes proliferation of lung squamous cell carcinoma by binding to miR-30d/e. J Exp Clin Cancer Res.

[30] (2020). Knockdown of PVT1 suppresses colorectal cancer progression by regulating MiR-106b-5p/FJX1 axis. Cancer Manag Res.

[31] (2021). Circ3823 contributes to growth, metastasis and angiogenesis of colorectal cancer: involvement of miR-30c-5p/TCF7 axis. Mol Cancer.

[32] (2019). Past, present, and future of circRNAs. EMBO J.

[33] (2021). Epigenetic regulation in fibrosis progress. Pharmacol Res.

[34] (2015). The anti-fibrotic effects and mechanisms of MicroRNA-486-5p in pulmonary fibrosis. Sci Rep.

[35] (2017). The role of miR-497-5p in myofibroblast differentiation of LR-MSCs and pulmonary fibrogenesis. Sci Rep.

[36] (2022). Circ-GGA3 promotes the biological functions of human lens epithelial cells depending on the regulation of miR-497-5p/SMAD4 axis. Biochem Biophys Res Commun.

[37] (2022). A novel MIR503HG/miR-497-5p/CCL19 axis regulates high glucose-induced cell apoptosis, inflammation, and fibrosis in human HK-2 cells. Appl Biochem Biotechnol.

[38] (2021). Emodin attenuates silica-induced lung injury by inhibition of inflammation, apoptosis and epithelial-mesenchymal transition. Int Immunopharmacol.

[39] (2019). Pirfenidone inhibits epithelial-mesenchymal transition and pulmonary fibrosis in the rat silicosis model. Toxicol Lett.

[40] (2014). E proteins in lymphocyte development and lymphoid diseases. Curr Top Dev Biol.

[41] (2009). E2A proteins maintain the hematopoietic stem cell pool and promote the maturation of myelolymphoid and myeloerythroid progenitors. Proc Natl Acad Sci U S A.

[42] (2021). Decreased expression of ATF3, orchestrated by β-catenin/TCF3, miR-17-5p and HOXA11-AS, promoted gastric cancer progression *via* increased β-catenin and CEMIP. Exp Mol Med.

[43] (2022). SHMT2 inhibition disrupts the TCF3 transcriptional survival program in Burkitt lymphoma. Blood.

[44] (2021). E2A Modulates stemness, metastasis, and therapeutic resistance of breast cancer. Cancer Res.

[45] (2019). E_2_-mediated EMT by activation of β-catenin/Snail signalling during the development of ovarian endometriosis. J Cell Mol Med.

[46] (2006). Overexpression of E2A proteins induces epithelial-mesenchymal transition in human renal proximal tubular epithelial cells suggesting a potential role in renal fibrosis. FEBS Lett.

[47] (2021). The NLRP3-inflammasome-caspase-1 pathway is upregulated in idiopathic pulmonary fibrosis and acute exacerbations and is inducible by apoptotic A549 cells. Front Immunol.

[48] (2020). Tanshinone IIA attenuates silica-induced pulmonary fibrosis *via* Nrf2-mediated inhibition of EMT and TGF-β1/Smad signaling. Chem Biol Interact.

