# Author Correction: *circPVT1* promotes silica-induced epithelial-mesenchymal transition by modulating the miR-497-5p/TCF3 axis

**DOI:** 10.7555/JBR.39.20250900

**Published:** 2025-03

**Authors:** Siyun Zhou, Yan Li, Wenqing Sun, Dongyu Ma, Yi Liu, Demin Cheng, Guanru Li, Chunhui Ni

**Affiliations:** 1 Department of Occupational Medical and Environmental Health, Key Laboratory of Modern Toxicology of Ministry of Education, Center for Global Health, School of Public Health, Nanjing Medical University, Nanjing, Jiangsu 211166, China; 2 Gusu School, Nanjing Medical University, Nanjing, Jiangsu 211166, China

Correction to: Journal of Biomedical Researchhttps://doi.org/10.7555/JBR.37.20220249, published on March 28, 2024.

The authors wish to correct the affiliation of the second author, Yan Li, to the Department of Occupational Medical and Environmental Health, Key Laboratory of Modern Toxicology of Ministry of Education, Center for Global Health, School of Public Health, Nanjing Medical University, Nanjing, Jiangsu 211166, China, because part of the research for this paper was conducted while Yan Li was affiliated with this institution.

