# Supplementary material for: circPVT1 promotes silica-induced epithelial-mesenchymal transition by modulating the miR-497-5p/TCF3 axis
Source: J Biomed Res. 2024 Mar 26;38(2):163–74. doi: 10.7555/JBR.37.20220249 (PMC11001589; doi:10.7555/JBR.37.20220249)
Supplement: Supplementary file 1 — Supplementary data to this article can be found online. [file jbr-38-2-163-S1.pdf]

Available online at www.jbr-pub.org.cn
Open Access at PubMed Central
Journal of Biomedical Research, 2024 0(000): 1–5
Supplementary Data
JBR

circPVT1 promotes silica-induced epithelial-mesenchymal transition by modulating the miR-497-5p/TCF3 axis

Siyun Zhou[1,△], Yan Li[2,△], Wenqing Sun[1,△], Dongyu Ma[1], Yi Liu[3], Demin Cheng[1], Guanru Li[1], Chunhui Ni[1,✉]

[1]*Department of Occupational Medical and Environmental Health, Key Laboratory of Modern Toxicology of Ministry of Education, Center for Global Health, School of Public Health, Nanjing Medical University, Nanjing, Jiangsu 211166, China;*
[2]*Biomedical Publications Center, Nanjing Medical University, Nanjing, Jiangsu 211166, China;*
[3]*Gusu School, Nanjing Medical University, Nanjing, Jiangsu 211166, China.*

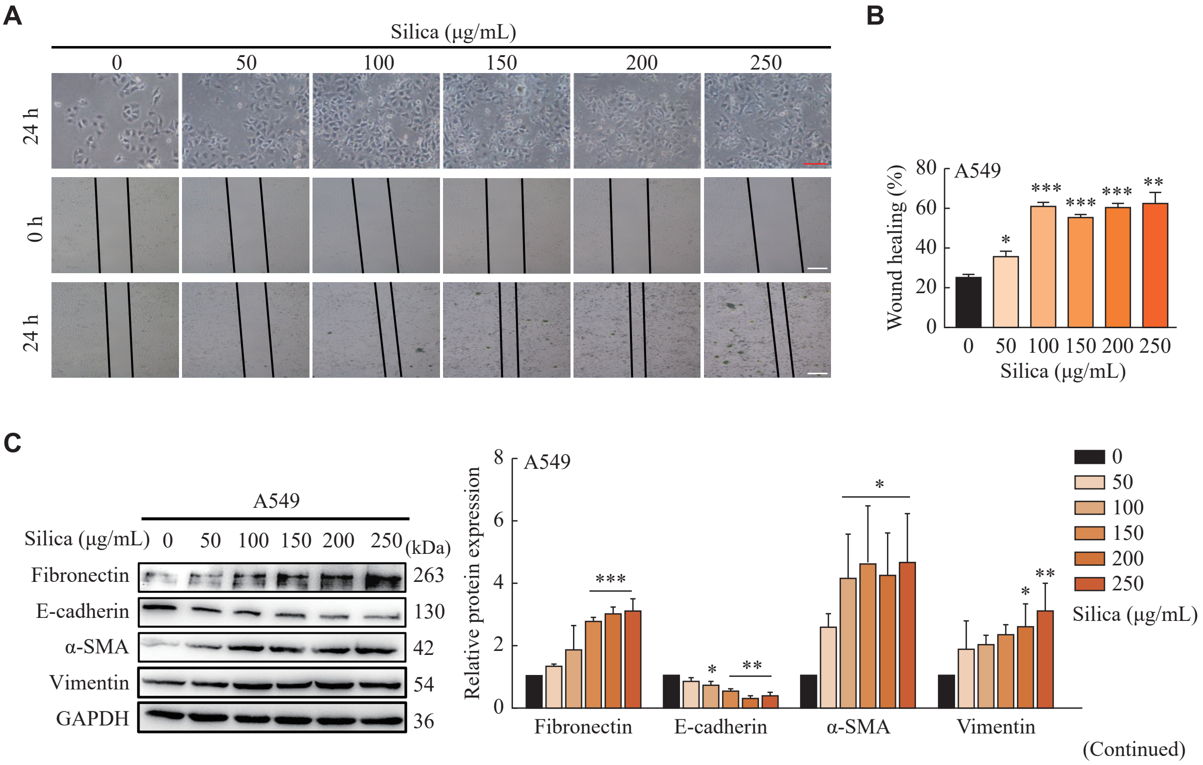

(Continued)

△These authors contributed equally to this work.

✉Corresponding author: Chunhui Ni, Department of Occupational Medical and Environmental Health, Key Laboratory of Modern Toxicology of Ministry of Education, Center for Global Health, School of Public Health, Nanjing Medical University, 101 Longmian Avenue, Nanjing, Jiangsu 211166, China. E-mails: chni@njmu.edu.cn and chninjmu@126.com.

Received: 30 November 2022; Revised: 22 May 2023; Accepted: 25 May 2023; Published online: 26 March 2024

CLC number: R135.2, Document code: A

The authors reported no conflict of interests.

This is an open access article under the Creative Commons Attribution (CC BY 4.0) license, which permits others to distribute, remix, adapt and build upon this work, for commercial use, provided the original work is properly cited.

© 2024 by Journal of Biomedical Research.

https://doi.org/10.7555/JBR.37.20220249

(Continued)

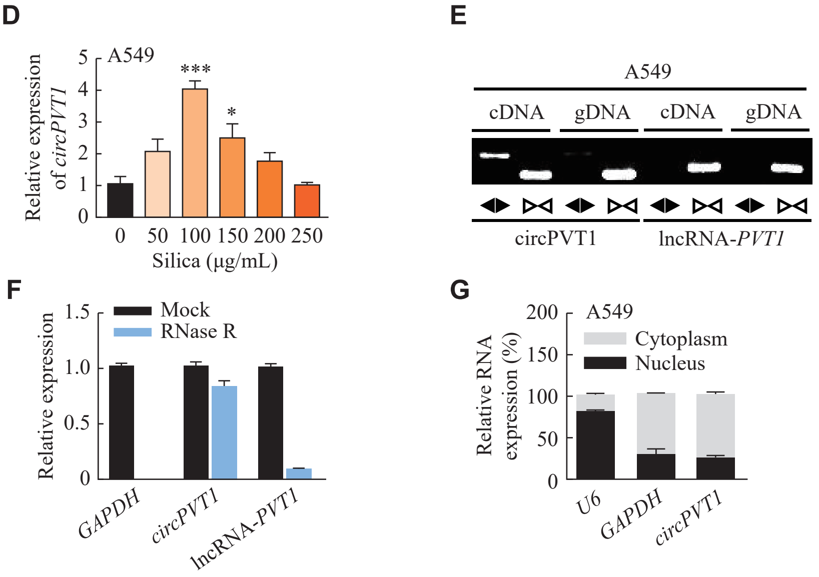

Supplementary Fig. 1 ***circPVT1*** **was involved in the silica-induced EMT process in A549 cells.** A549 cells were treated with 0, 50, 100, 150, 200, and 250 μg/mL silica particles for 24 h. A. Changes in cell morphology and representative images of wound healing assay. Scale bar, 25 μm (upper) and 250 μm (middle and lower). B: Quantitative analysis of the wound healing assay. C: Western blotting and quantitative analysis of fibronectin, E-cadherin, α-SMA, and vimentin expression levels. D: Quantitative reverse transcription-PCR (qRT-PCR) detection of *circPVT1*. E: *circPVT1* and linear lncRNA-*PVT1* were amplified by divergent and convergent primers in complementary DNA (cDNA) and genomic DNA (gDNA). The black triangle pair indicates divergent primers, and the white triangle pair indicates convergent primers. F: qRT-PCR was used to detect the expression of *GAPDH*, *circPVT1*, and long non-coding RNA (lncRNA)-*PVT1* after RNase R digestion. G: qRT-PCR detection of *circPVT1* expression in nuclear and cytoplasm of A549 cells. *U6* and *GAPDH* were used as nuclear and cytoplasmic controls, respectively. Data are presented as mean ± standard deviation from three experiments. Statistical analyses were performed by Student's *t*-test. $^{*}P < 0.05$ and $^{***}P < 0.001$ *vs.* the 0 μg/mL silica group. Abbreviations: EMT, epithelial-mesenchymal transition; α-SMA, α-smooth muscle actin; GAPDH, glyceraldehyde 3-phosphate dehydrogenase.

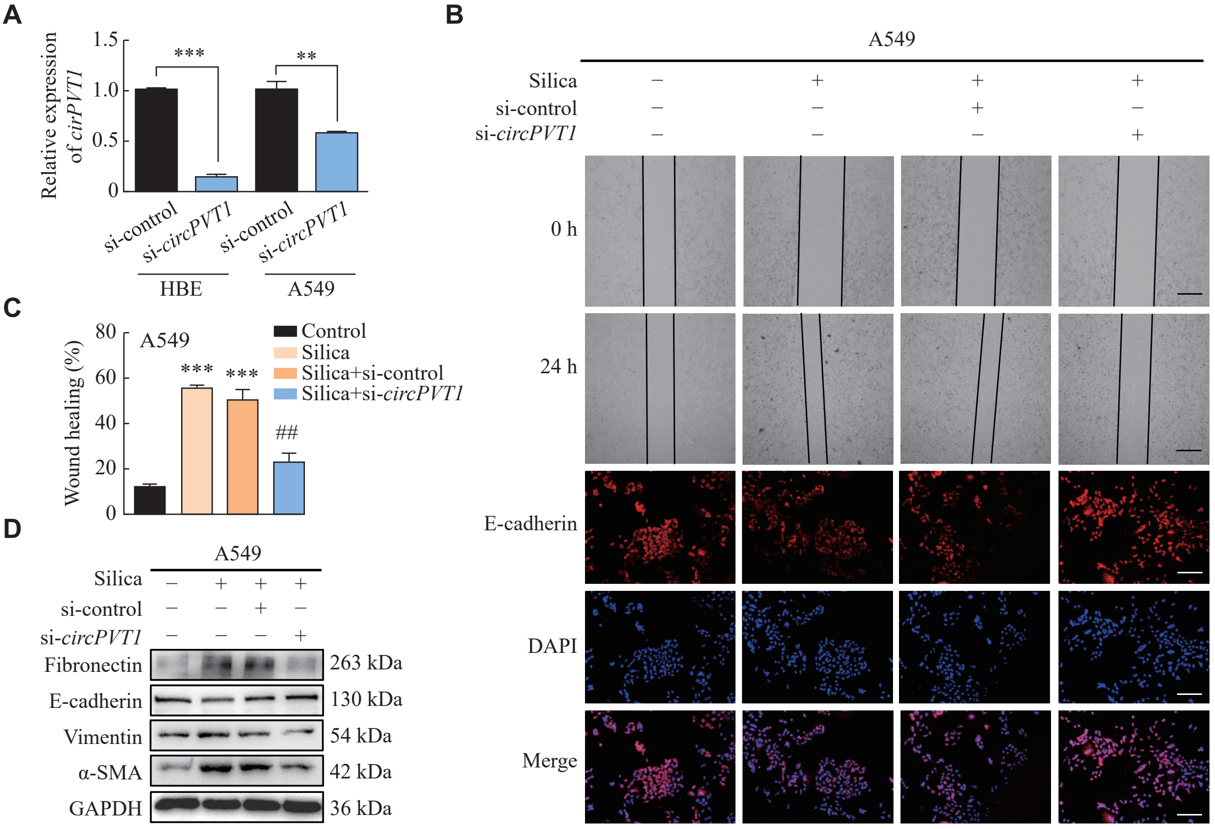

(Continued)

(Continued)

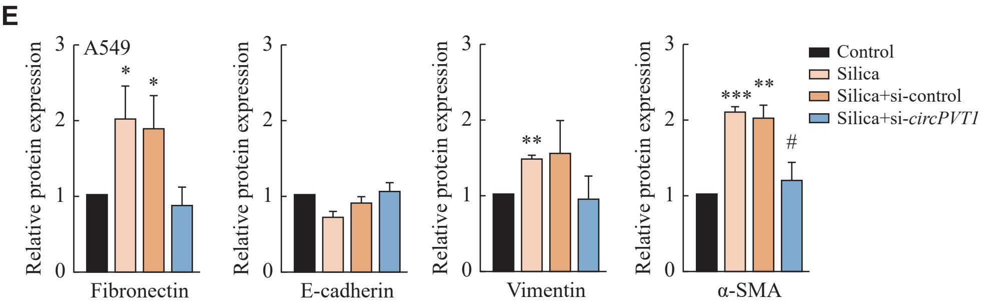

***Supplementary Fig. 2* The knockdown of *circPVT1* attenuated the silica-induced EMT process in A549 cells.** A: Quantitative reverse transcription-PCR detection to evaluate the efficiency of *circPVT1*-small interfering RNA (si-*circPVT1*) in HBE and A549 cells. $^{**}P < 0.05$ and $^{***}P < 0.001$ *vs.* the si-control group by student's *t*-test. A549 cells were transfected with si-*circPVT1* or si-control for 24 h, and then treated with 100 μg/mL silica for 24 h. B: Representative images of wound healing assay and immunofluorescence staining of E-cadherin. C: Quantitative analysis of wound healing assay. D and E: Protein levels of fibronectin, E-cadherin, α-SMA, and vimentin were detected by Western blotting. Data are presented as mean ± standard deviation from three independent experiments. Statistical analyses were performed by one-way ANOVA followed by Tukey's post hoc tests for multiple comparisons. $^{*}P < 0.05$, $^{**}P < 0.01$, and $^{***}P < 0.001$ *vs.* the control group. $^{\#}P < 0.05$ and $^{\#\#}P < 0.01$ *vs.* the silica + si-control group. Abbreviations: EMT: epithelial-mesenchymal transition; α-SMA: α-smooth muscle actin; HBE, human bronchial epithelial.

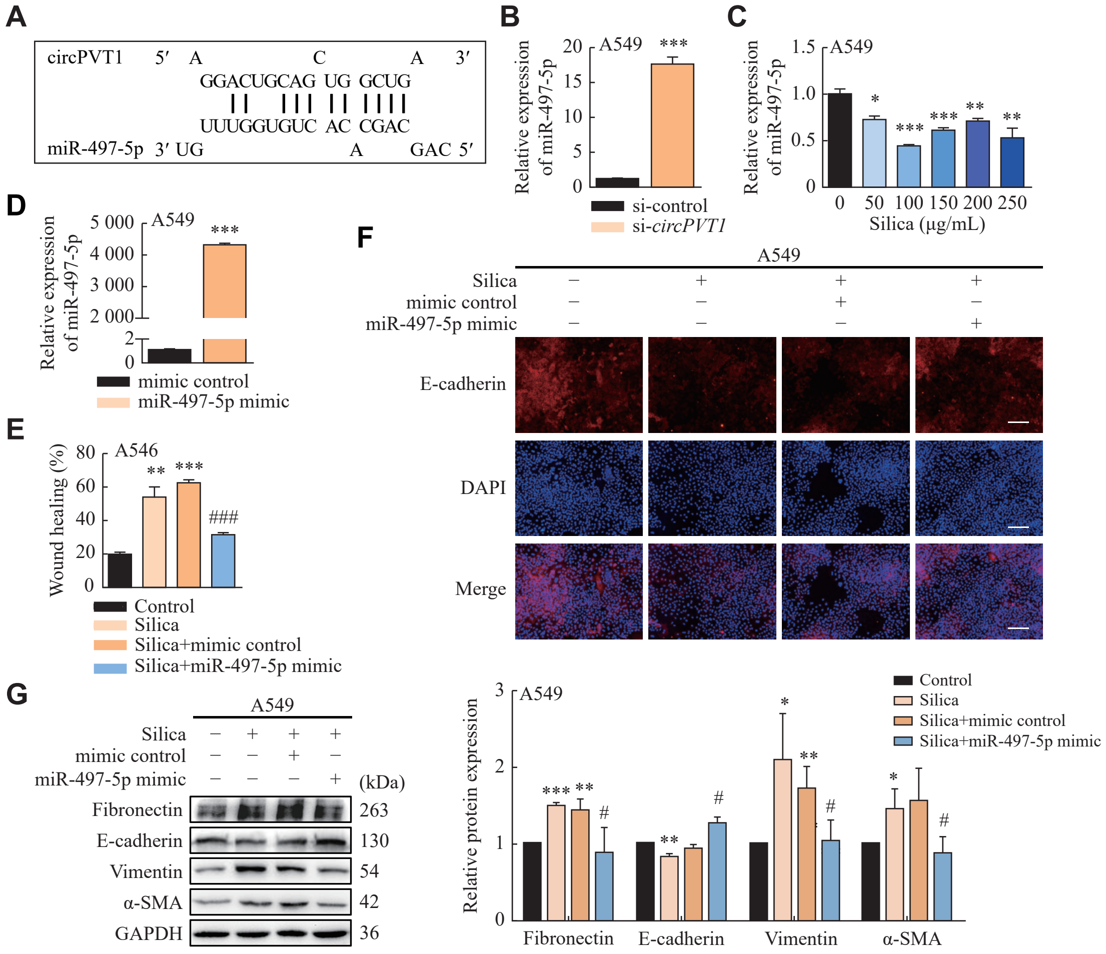

***Supplementary Fig. 3* *circPVT1*/miR-497-5p axis was involved in the silica-induced EMT process in A549 cells.** A: A potential binding site of *circPVT1* on miR-497-5p. B: Quantitative reverse transcription-PCR (qRT-PCR) detection of miR-497-5p after silencing *circPVT1* for 24 h in A549 cells. $^{***}P < 0.001$ *vs.* the si-control group by student's *t*-test. C: qRT-PCR detection of miR-497-5p after being treated with different doses of silica particles for 24 h in A549 cells. $^{*}P < 0.05$, $^{**}P < 0.01$, and $^{***}P < 0.001$ *vs.* the 0 μg/mL silica group by one-way ANOVA with Dunnett's tests. D: A549 cells were transfected with miR-497-5p or mimic control for 24 h. qRT-PCR detection to test the over-expression efficiency of miR-497-5p mimic. $^{***}P < 0.001$ *vs* the mimic control group by student's *t*-test. A549 cells were transfected with miR-497-5p mimic or mimic control for 24 h, and then treated with 100 μg/mL silica for 24 h. E: Quantitative analysis of wound healing assay. F: Immunofluorescence staining of E-cadherin. G: The protein levels of fibronectin, E-cadherin, α-SMA, and vimentin were detected by Western blotting followed by corresponding quantitative analysis. $^{*}P < 0.05$, $^{**}P < 0.01$, and $^{***}P < 0.001$ *vs.* the control group and $^{\#}P < 0.05$ *vs.* the silica + mimic control group by one-way ANOVA with Tukey's post hoc tests. Data are presented as mean ± standard deviation from three independent experiments. Abbreviations: EMT, epithelial-mesenchymal transition; α-SMA, α-smooth muscle actin; GAPDH, glyceraldehyde 3-phosphate dehydrogenase.

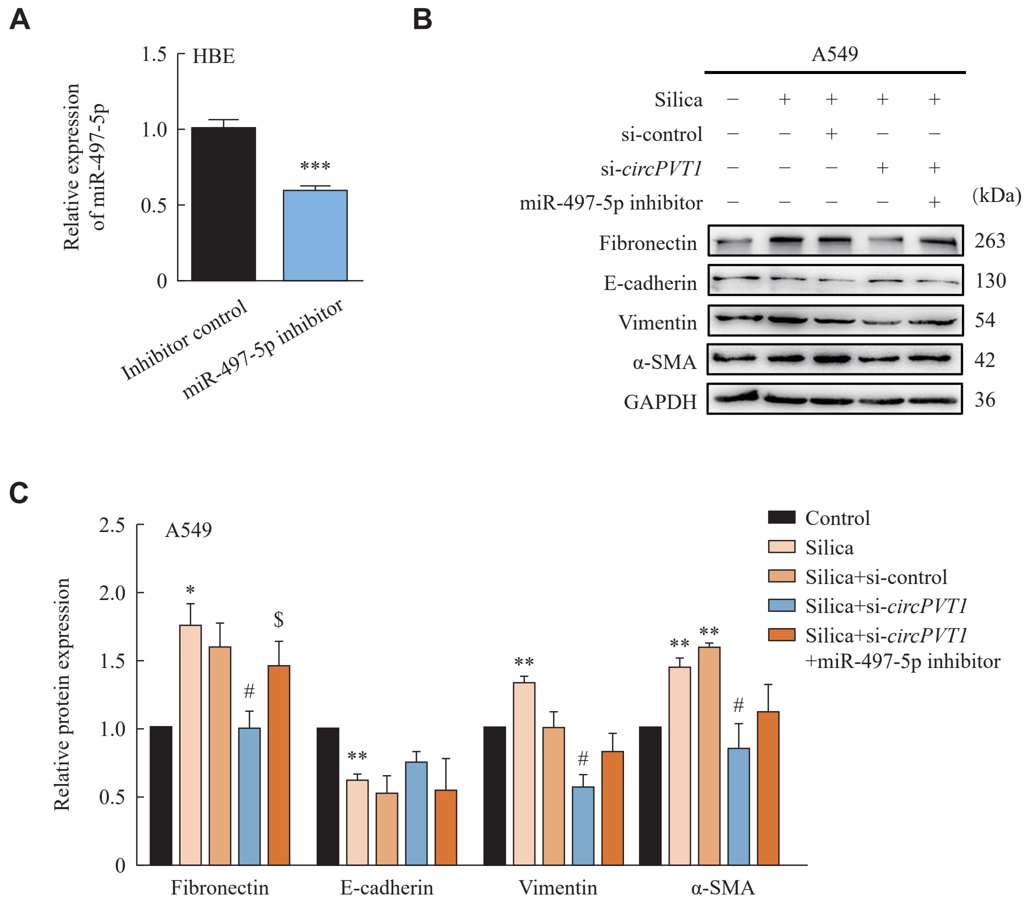

Supplementary Fig. 4 **miR-497-5p inhibitor abolished the anti-fibrosis and anti-EMT effect of *circPVT1* siRNA.** A: HBE cells were transfected with miR-497-5p inhibitor or inhibitor control for 24 h. Quantitative reverse transcription-PCR detection to test the efficiency of miR-497-5p inhibitor in HBE cells. $^{***}P < 0.001$ *vs.* the inhibitor control group by student's *t*-test. B and C: A549 cells were transfected with *circPVT1*-small interfering RNA (si-*circPVT1*) alone or plus miR-497-5p inhibitor for 24 h, and then treated with 100 μg/mL silica for 24 h. The protein levels of fibronectin, E-cadherin, α-SMA, and vimentin were detected by Western blotting followed by quantitative analysis. Statistical analyses were performed by one-way ANOVA with Tukey's post hoc tests. $^{*}P < 0.05$, $^{**}P < 0.01$, and $^{***}P < 0.001$. Data are presented as mean ± standard deviation from three independent experiments. Abbreviations: EMT, epithelial-mesenchymal transition; α-SMA, α-smooth muscle actin; HBE, human bronchial epithelial; GAPDH, glyceraldehyde 3-phosphate dehydrogenase.

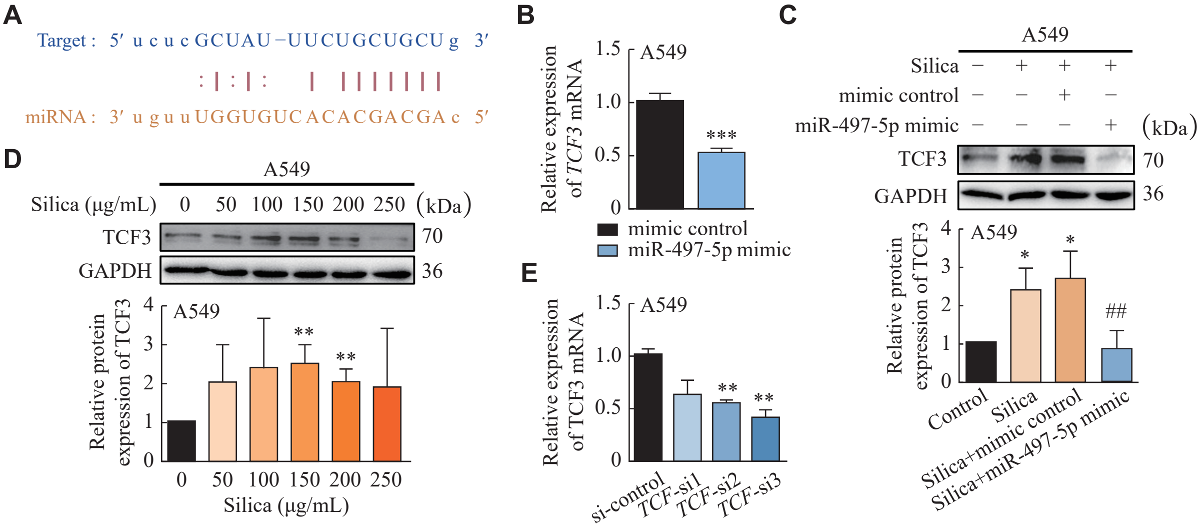

(Continued)

(Continued)

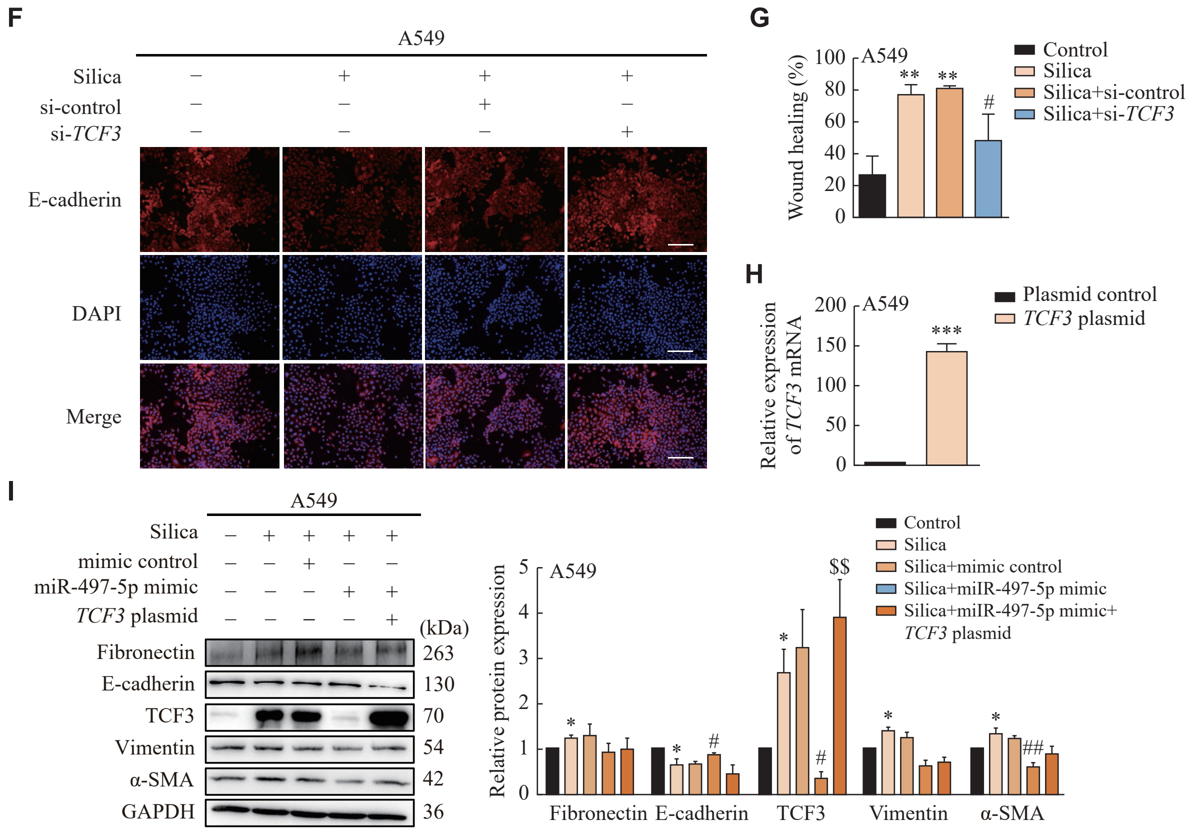

Supplementary Fig. 5 **miR-497-5p inhibited the EMT process *via* down-regulating TCF3 in A549 cells.** A: A potential binding site of miR-497-5p on the 3′ untranslated region of *TCF3* mRNA. B: Quantitative reverse transcription-PCR (qRT-PCR) detection of *TCF3* after being treated with miR-497-5p mimic. $^{***}P < 0.001$ *vs.* the mimic control group by student's *t*-test. C: A549 cells were transfected with miR-497-5p mimic or control for 24 h, and then treated with 100 μg/mL silica for 24 h. The representative images of Western blotting and quantitative analysis on TCF3 protein. $^{*}P < 0.05$ *vs.* the control group and $^{\#\#}P < 0.01$ *vs.* the silica + mimic control group by one-way ANOVA with Tukey's post hoc tests. D: Western blotting and quantitative analysis of TCF3 after being treated with different doses of silica particles. $^{**}P < 0.01$ *vs.* the 0 μg/mL silica group by one-way ANOVA with Dunnett's post hoc tests. E: qRT-PCR detection of *TCF3* after TCF3-small interfering RNA (si-*TCF3*) or si-control treatment. $^{**}P < 0.01$ *vs.* the si-control group by one-way ANOVA with Dunnett's post hoc tests. A549 cells were exposed to si-*TCF3* or si-control for 24 h, and then treated with 100 μg/mL silica for 24 h. F: Immunofluorescence staining of E-cadherin. G: Quantitative analysis of wound healing assay. $^{**}P < 0.01$ *vs.* the control group and $^{\#}P < 0.05$ *vs.* the silica + si-control group by one-way ANOVA with Tukey's post hoc tests. H: qRT-PCR detection of *TCF3* to test the over-expression efficiency of *TCF3* plasmid. $^{***}P < 0.001$ *vs.* the plasmid control group by student's *t*-test. I: A549 cells were transfected with miR-497-5p mimic alone or plus *TCF3* plasmid for 24 h, and then treated with 100 μg/mL silica for 24 h. The protein levels of fibronectin, E-cadherin, α-SMA, and vimentin were detected by Western blotting and quantitative analysis. Statistical analyses were performed by one-way ANOVA followed by Tukey's post hoc tests for multiple comparisons. $^{*}P < 0.05$ *vs.* the control group. $^{\#}P < 0.05$ and $^{\#\#}P < 0.01$ *vs.* the silica + mimic-NC group. $^{\$\$}P < 0.01$ *vs.* the silica + miR-497-5p mimic group. Data are presented as mean ± standard deviation from three independent experiments. Abbreviations: EMT, epithelial-mesenchymal transition; α-SMA, α-smooth muscle actin; TCF3, transcription factor 3; GAPDH, glyceraldehyde 3-phosphate dehydrogenase.
